# The Analysis of Multiple Genome Comparisons in Genus *Escherichia* and Its Application to the Discovery of Uncharacterised Metabolic Genes in Uropathogenic *Escherichia coli* CFT073

**DOI:** 10.1155/2009/782924

**Published:** 2010-03-02

**Authors:** William A. Bryant, Preben Krabben, Frank Baganz, Yuhong Zhou, John M. Ward

**Affiliations:** ^1^Advanced Centre for Biochemical Engineering, University College London, Torrington Place, London WC1E 7JE, UK; ^2^Structural and Molecular Biology, Darwin Building, University College London, Gower Street, London WC1E 6BT, UK; ^3^Greenbiologics Ltd, Unit 45A, Milton Park, Abingdon, Oxfordshire, OX14 4RU, UK

## Abstract

A survey of a complete gene synteny comparison has been carried out between twenty fully sequenced strains from the genus *Escherichia* with the aim of finding yet uncharacterised genes implicated in the metabolism of uropathogenic strains of *E. coli* (UPEC). Several sets of adjacent colinear genes have been identified which are present in all four UPEC included in this study (CFT073, F11, UTI89, and 536), annotated with putative metabolic functions, but are not found in any other strains considered. An operon closely homologous to that encoding the L-sorbose degradation pathway in *Klebsiella pneumoniae* has been identified in *E. coli* CFT073; this operon is present in all of the UPEC considered, but only in 7 of the other 16 strains. The operon's function has been confirmed by cloning the genes into *E. coli* DH5*α* and testing for growth on L-sorbose. The functional genomic approach combining in silico and in vitro work presented here can be used as a basis for the discovery of other uncharacterised genes contributing to bacterial survival in specific environments.

## 1. Introduction

Uropathogenic *E. coli* (UPEC) are the causal agents of 80% of all community-acquired urinary tract infections (UTIs) [1]. The ability of UPEC to colonise the urinary tract has been studied in depth in terms of virulence factors such as pili and hemolysins [2]; however, the ability to utilise the metabolites available in this environment has not been fully investigated to date. While studies of growth rates in urine have been conducted (for instance by Gordon and Riley [3]), it has never been established exactly what is used as the primary carbon source (or if there is a single one) for growth of UPEC, and which genes enable the utilisation of this carbon source. There has been discussion of metabolic genes contributing to uropathogenicity, considering two UPEC: CFT073 and 536 [4]. D-serine has been posited as a potential carbon and nitrogen source due to the presence of D-serine catabolic genes in these 2 UPEC, though the requirement for these genes or their involvement in the course of a UTI has yet to be investigated.

The use of multiple genome analysis in research has been reviewed elsewhere [5], and several tools for genome comparisons are available, as well as software applications for their analysis. The most prominent tool is BLAST [6] for whole genome analyses. The set of genus *Escherichia* genome sequences has already been used to compare regulatory networks [7] and to investigate the genetic basis of pathogenesis in enterotoxigenic *E. coli* [8]. Sequencing projects (such as [4, 9]) have used up to three complete genome sequences for genomic comparisons, though this is only a small subset of the *Escherichia* genome sequences currently available. Recently Rasko et al. [10] have used a BLAST score ratio (BSR) technique to compare the gene contents of 17 *E. coli* genome sequences which has identified fewer pathovar-specific genes than might be expected.

The potential for taking advantage of the large number of already available and imminent genome sequences in the genus *Escherichia* is great, when combined with well-targeted experimentation. While there is no certainty from purely computational approaches that a gene is necessary or helpful in a particular environment, its presence in bacteria which thrive in that environment and its absence in bacteria which do not represent evidence for pressure to retain the gene, therefore, its function aids the persistence of the bacteria in that niche. In this study the genome sequence of *E. coli* CFT073 has been compared to 19 other complete genome sequences of bacteria of the genus *Escherichia* using a synteny approach to infer which genes are present in or absent from the other strains. Table 1 shows a list of these strains which includes four UPEC and three *Shigella* strains.

Genes in CFT073 which according to their GenBank entries [11] are metabolic genes, but which do not have a specific functional assignment (e.g., gene c4985 is annotated as “Putative sorbose PTS component”), or genes of unknown function which form sets of adjacent colinear genes with those partially characterised metabolic genes, have been investigated to determine their relative occurrences in UPEC and the other strains. Some of those sets of genes more often found in UPEC than the other strains used in this study have been investigated to further elucidate their function.

One putative operon has been experimentally verified as an L-sorbose utilisation operon, after having been identified as being present more frequently in UPEC than in the other strains considered. Lehmacher and Bockemühl [12] showed in a study of 266 strains from the collection of the Institut für Hygiene und Umwelt that L-sorbose utilisation varies widely over differing pathotypes of *E. coli* and *Shigella*, from 14 of the 15 *E. coli* isolates associated with neonatal meningitis to a complete lack of utilisation by *Shigella* (of the 26 tested in the study). Although no UPEC were included, 67% of the EPEC and EAEC strains tested utilised L-sorbose.

## 2. Materials and Methods

### 2.1. Multiple Genome Comparisons

The CFT073 genome [9] was compared gene-by-gene with the genomes of the nineteen other strains listed in Table 1 using TBLASTN [6], and a synteny result was obtained by manual inspection of each individual gene-nucleotide comparison. Homology as a percentage identity from TBLASTN was combined with the position and homology of nearest neighbours and overall position in the genome to infer synteny conservation. The use of BLAST scores to determine gene conservation is well established (such as the BSR technique [10]), and this process was refined by adding a neighbour-dependent analysis to determine gene synteny. For sets of two or more genes in a similar position on two genomes, retention of function of each gene was inferred by identity with a cut-off of 90% over the whole length of each gene. Single genes in similar positions on the two genomes were inferred as conserved only if their mutual identity was above 95% over their whole length.

MG1655 does not grow in urine, whereas CFT073 does [13]. Some metabolic genes in CFT073 that are not present in MG1655 may confer a fitness advantage in the urinary tract due to better ability to use available substrates for growth. Genes present in CFT073 but not in MG1655 were therefore studied to look for such metabolic genes. This produced an initial list of candidate genes that could be implicated in the metabolism of CFT073 in the urinary tract, but have not yet been characterised. This set of genes was inspected manually for those genes which appeared to have a metabolic function from their GenBank annotation [11], but without a definite, specific biological function, henceforth referred to as putatively metabolic genes (PMGs).

Where there was a PMG surrounded by uncharacterised genes, this region of the genome sequence was viewed using the NCBI's Sequence Viewer 2.1 and all adjacent genes transcribed in the same direction as the PMG and less than 100 base pairs separated were labelled as part of a Set of Adjacent Colinear genes (SAC) and included in the analysis. The algorithm for inferring whether a SAC was present in each of the *Escherichia* genomes considered was as follows: where the SAC was greater than 3 genes in length it was considered present if at most one of the genes was absent according to the synteny comparison; where there were 3 or fewer genes, all genes had to be present to conclude that the set of genes was present.

BLASTp was then used on each gene in the SACs against the full nonredundant database of Genbank [14] to try to find homologies to already annotated genes. Where homologs could not be found, protein domain similarities were sought using Pfam [15] and SEED (http://theseed.uchicago.edu/FIG/index.cgi) in an endeavour to elucidate their function. Further, the NCBI's Conserved Domain Database was searched for conserved domains. The results of the Conserved Domain Database searches can be seen in Supplementary Table 2 in supplementry matarial available online at doi:10.1155/2009/782924.

A phylogenetic comparison between the putative L-sorbose operons from those sequenced bacteria of the genus *Escherichia*, using *Klebsiella pneumoniae* (GI: 150953431) and *Klebsiella oxytoca* (see acknowledgments for reference) as an outgroup, was conducted using ClustalX [16]. BLAST was used to extract the putative L-sorbose operons for most of the bacteria, but Artemis [17] was required to extract the relevant parts of both the *Klebsiella* strains and F11, which do not yet have complete single contig genome sequences.

### 2.2. Experimental Verification of Putative Functions of Genes c4981 to c4987 from *Escherichia coli* CFT073

Escherichia coli strain DH5*α* was used as the test host for L-sorbose growth as it is known not to grow on L-sorbose and is designed to be highly competent. The genotype of DH5*α* is: F^−^ *fhuA2 *Δ*(lacZ-argF)U169* *ϕ* *80dlacZ *Δ*M15 endA1 hsdR17 deoR nupG thi-1 supE44 gyrA96 relA1 recAI phoA* *λ* ^−^.

**Figure 1:**
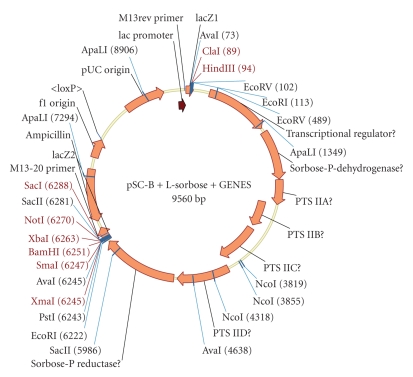
Structure of the pSC-B plasmid with the putative L-sorbose operon insert from CFT073.

The putative L-sorbose operon identified in CFT073 was excised using the forward and reverse primers 5′-GCCAGCGACATGCAGAGTTAAGTAGCGCGA-3 and 5′-AAATCTCCTGTAAAACGCGGAATATACC-3, respectively. The consequent 7.5 kb fragment was amplified using these primers with Phusion DNA polymerase (Finnzyme) and cloned into the pSC-B plasmid (Stratagene). The consequent construct was transformed into DH5*α* and bacteria with a correctly inserted plasmid were selected by blue/white screening on 100 *μ*g ml^−1^ ampicillin, 40 *μ*g ml^−1^ X-Gal, 20 *μ*g ml^−1^ IPTG plates and were picked off and spread onto a Nutrient Broth 2 (Oxoid) plate with 100 *μ*g ml^−1^ ampicillin to produce single colonies. The insertion and orientation were confirmed by sequencing from the M13 primers to be as shown in Figure 1 and the plasmid was named pQR793.

One colony was then picked from the Nutrient Broth 2 plate and grown in 5 mL of a modified liquid M6 medium (5·20 gl^−1^ (NH_4_)_2_ SO_4_, 3.86 gl^−1^ NaH_2_PO_4_·H_2_O, 4.03 gl^−1^ KCl, 4.16 gl^−1^ Citric Acid, 1.04 gl^−1^ MgSO_4_·7H_2_O, 0.25 gl^−1^ CaCl_2_·2H_2_O, 20.6 mgl^−1^ ZnSO_4_·7H_2_O, 27.2 mgl^−1^ MnSO_4_·4H_2_O, 8.1 mgl−1 CuSO_4_·5H_2_O, 4.2 mgl^−1^ CoSO_4_·7H_2_O, 100.6 mgl^−1^ FeCl_3_·6H_2_O, 0.3 mgl^−1^ H_3_BO_3_, 0.2 mgl^−1^ Na_4_MoO_4_·2H_2_O adjusted to pH 7.3 by NaOH) with 1% glucose, 0.001% thiamine (a requirement for DH5*α* to grow) and 100 *μ*g ml^−1^ ampicillin; shaken at 150 rpm, 37°C overnight, then inoculated into two 100 mL shake flasks, one containing the same medium composition as the starter culture and the other containing 1% L-sorbose instead of glucose. Simultaneously DH5*α* with an empty pUC19 plasmid and CFT073 were grown in the same manner as negative and positive controls, respectively, (no ampicillin was used in the growth of CFT073 as it is not ampicillin resistant and selection is not required).

These flasks were shaken at 150 rpm at 37°C for 145 hours and samples were taken at intervals to measure optical density of the samples at wavelength 600 nm. Each culture was repeated twice to confirm the result growth or lack thereof.

## 3. Results and Discussion

### 3.1. Multiple Genome Comparison

The set of genome sequences used in this study will be referred to as the EGSS (*Escherichia* genome sequence set); the results of the complete synteny comparison of the genome of CFT073 against the rest of the EGSS can be seen in Supplementary Table 1.

**Figure 2:**
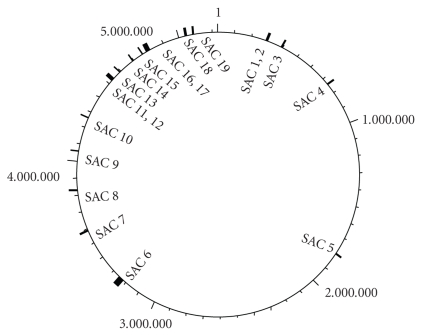
Positions of the SACs identified in the genome sequence of *E. coli* CFT073, as labelled in Table 2.

Overall 133 PMGs were inferred; they can be seen in Supplementary Table 2, along with the results of the NCBI Conserved Domain Search, and are summarised in Table 2. All of the individual genes marked with putative metabolic functions in the CFT073 genome sequence were positioned adjacent to genes transcribed in the same sense, so the sets of adjacent colinear genes were included in the investigation, a summary of which can be seen in Table 2, and positions of which in the CFT073 genome can be seen in Figure 2. It should be emphasised that the criterion for consideration of these genes was only that the genes marked with putative functions be absent from *E. coli* K-12 MG1655, without any further consideration of whether they would specifically be useful in the lifecycle of CFT073. 9 of these genes are present in all of and only in the UPEC and 49 genes are among those identified by Lloyd et al. [18], which compared the gene content of 7 additional UPEC and 2 different faecal strains of *E. coli* by comparative genomic hybridisation against CFT073 to find those genes unique to uropathogens.

The synteny comparison shows several characteristics indicating a higher prevalence of the genes from CFT073 in the other UPEC than in the other strains. It is worth noting that the sets of adjacent colinear genes identified in this study are not in general parts of the large pathogenicity islands identified in CFT073 by others [9, 18], with the exception of those marked with an asterisk in Table 2.

SAC No. 6 (as labelled in Table 2) is within a large pathogenicity island, PAI-CFT073-*metV* (according to the nomenclature set out by Lloyd et al. [18]), in which the SAC is restricted to the area c3405 to c3410. These genes are retained in all the UPEC, in *E. coli* E2348, and in *E. coli* SMS-3-5, but not in any other strain. Retention of the *SorCDFBAME* genes (SAC No. 15) in some of the EAEC and EPEC and all of the *Shigella* is consistent with the findings of Lehmacher and Bockemühl [12] who despite the negative phenotype showed that they retain the DNA for many of these genes.

It was found that 121 of the 133 PMGs identified are present in the same position in all four UPEC; these include the putative genes for L-sorbose degradation. The only SAC not present in any of the UPEC other than CFT073 is No. 1. Those found in the same place in all UPEC, but in none of the other strains, are 8, 12, and 19. The SACs identified here have a tendency to be present or absent as a whole, rather than on a gene-by-gene basis.

An investigation of each of the PMGs was conducted using BLAST, the SEED tool, Pfam, and the NCBI Conserved Domain Database, in an attempt to identify putative functions for all the genes. SAC No. 8 (genes c4013 to c4018) has several genes annotated putatively already, c4015 to c4017 as part of a ribose ABC transporter, and c4018 as a tagatose 1,6-diphosphate aldolase. The hypothetical genes bear similarities to other sugar metabolism encoding genes: c4013 to a dehydrogenase and c4014 to a sugar kinase, possibly a fructokinase. These genes could encode enzymes for the uptake and catabolism of a 5- or 6-carbon sugar or sugar derivative.

Since there is a limited number of carbon sources present in urine—predominantly urea, uric acid, and creatinine (and L-sorbose in small amounts)—it might seem plausible that the sets of genes identified here may encode proteins for the utilisation of these chemicals. However, an analysis of the genes investigated here has so far failed to find any good matches to known metabolic pathways for these three carbon containing compounds. UPEC are not confined to using the small molecule carbon sources in urine; they adhere primarily to the epithelial cells in the urinary tract so potentially the mucus produced by these cells could be used to fulfil the metabolic needs of UPEC.

The benefit of D-serine utilisation genes for UPEC has been suggested [4]; so these genes were investigated in the UPEC included in this study to assess their relative prevalence. It was noted that according to the initial synteny comparison (Supplementary Table 1), these genes are not present in *E. coli* F11 or 536. However, there are D-serine utilisation genes in both F11 and 536 at an alternative position in the genome identified by Brzuszkiewicz et al. [4]. Moreover, UTI89 also has D-serine utilisation genes in this alternative position on its genome as well as those found in this study. None of the non-UPEC in the EGSS have this alternative operon, which is characterised by a particularly large intergenic region (~1 kb) adjacent to it, conserved between F11, 536 and UTI89. This D-serine utilisation operon is therefore unique within the strains considered here to the UPEC.

### 3.2. L-sorbose In Silico Analysis

L-sorbose [19] can be present in urine (and in the gastrointestinal tract). It can be seen from Table 2 that it is predominantly the UPEC and *Shigella* strains which contain the operon enabling use of L-sorbose as a carbon source.

The putative L-sorbose operon in CFT073 was compared to that of *Klebsiella pneumoniae* using BLASTp. Both of these strains are in the family *Enterobacteriaceae* and the function of *Klebsiella*'s operon has been experimentally verified [20]. The results of this comparison are shown in Table 3, which shows identity above 90% for all but one of the genes.

**Figure 3:**
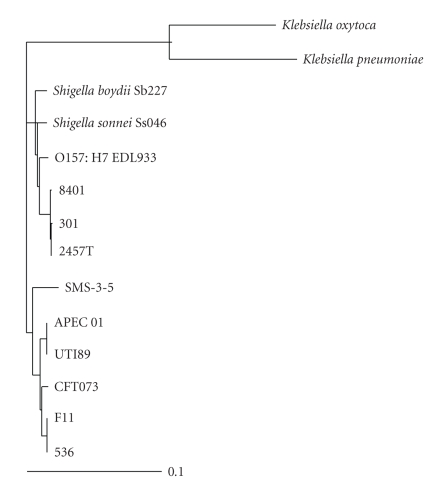
Phylogenetic tree of L-sorbose operons (both confirmed and putative) in the genus *Escherichia* and in two *Klebsiella* strains. The scale is in units of substitutions per site. Unless otherwise stated, the strain is *Escherichia coli*.

The ClustalX comparison of the putative sorbose operons of 13 strains of bacteria produced the phylogenetic tree shown in Figure 3. These bacteria are all of genus *Escherichia* (except the *Klebsiella* strains) and include several *Shigella* strains, *Shigella boydii* Sb227 (Accession Number: CP000036.1) and *Shigella sonnei* Ss046 (Accession Number: CP000038.1), not used in the full genome analysis because their genomes are not completely sequenced. The operons were located in the genome sequence thus far generated for them and extracted to compare to the others in the EGSS. Also included is the inferred L-sorbose operon from Avian Pathogenic *Escherichia coli* O1:K1:H7 (APEC 01) [21] (Accession Number: CP000468.1), which clusters with the UPEC, separate from the enterohaemorrhagic *E. coli* (EHEC) and *Shigella* strains. This is also true of the environmental *E. coli* SMS-3-5. The EHEC and *Shigella* L-sorbose operons are grouped together.

### 3.3. Experimental Verification of Function of Genes c4981 to c4987 from *Escherichia coli* CFT073

**Figure 4:**
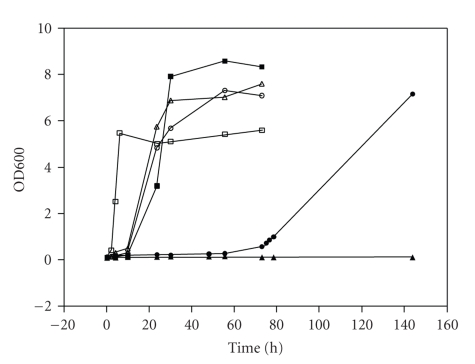
Growth curves for DH5*α* containing plasmid pQR793, compared to CFT073 and DH5*α* with an empty pUC-19 plasmid. DH5*α* with pQR793 is represented by ∘ and •, DH5*α* with pUC19 by Δ and ▴ and CFT073 by □ and ▪ where empty symbols represent growth on glucose and filled symbols represent growth on L-sorbose. Where duplicate samples were taken, readings varied by less than 0.01 OD_600_ units using a CO8000 Cell Density Meter (WPA).

The genes c4981 to c4987 were successfully cloned into the pSC-B plasmid and confirmed by sequencing as being oriented as shown in Figure 1. The growth of the three strains in minimal media containing either glucose or L-sorbose as sole carbon source can be seen in Figure 4. The negative control, DH5*α* with pUC19, was unable to utilise this carbon source. DH5*α* containing the pQR793 plasmid grew using L-sorbose as the sole carbon source.

The functions of genes c4981 to c4987 from *Escherichia coli* CFT073 have therefore been confirmed as those encoding a pathway for the utilisation of L-sorbose as a sole carbon source. The nucleotide sequence identity of between this operon (along its entire length) and the operon identified and experimentally characterised in *Klebsiella pneumoniae* [22] is 78%. Table 3 shows that all but one of the protein amino acid sequence differences between corresponding genes in these two operons are above 90%. These high similarities imply that not only do the two operons both utilise L-sorbose, but that the same pathway is used by both. The phylogenetic analysis of the L-sorbose operon conducted as part of this research shows how this operon fits in the phylogeny of L-sorbose operons in the genus *Escherichia*. The Glucitol-6-phosphate dehydrogenase found in *Klebsiella pneumoniae* has been shown to be temperature sensitive [20], which might account for the long lag phase and slow growth of DH5*α* containing the plasmid.

## 4. Conclusions

Multiple genome sequence analysis has been used to identify several sets of adjacent colinear genes in *E. coli* CFT073 that are not present in *E. coli* MG1655 and might be implicated in metabolism in the human urinary tract. Specifically a previously incompletely annotated operon encoding proteins involved in L-sorbose catabolism have been identified and experimentally confirmed encompassing genes c4981 to c4987 of the genome of uropathogenic *Escherichia coli* strain CFT073. The sets of genes from CFT073 found solely in UPEC include an arginine metabolic operon, which has previously been implicated in UPEC fitness in the urinary tract. The use of such sets of genomic data will become increasingly important as the rate of sequencing increases while experimental verification of gene function lags considerably behind. Although elucidation of novel gene function cannot be done purely through comparative genomics, it can aid searches for important genes, not necessarily previously characterised in other species or strains.

## Supplementary Material

The supplementary spreadsheet file contains two worksheets. Supplementary Table 1 shows a list of genes in *E. coli* CFT073, referred to by both GI number (from Genbank) and c number (from the sequencing project), along with a gene label where this has been established. Each of the following columns indicates the presence or absence (by a 1 or 0 respectively) of that gene in the organism indicated at the top of the column, according to the synteny comparison undertaken in the accompanying paper. The three organisms highlighted in grey are the other uropathogenic *E. coli* included in the comparison.
Supplementary Table 2 lists the genes inferred in the acompanying paper to be candidates for metabolic functions that have not been fully characterised. Along with GI number and c number is the name given to the gene in the genome sequencing project. Presence or absence of each gene in the other *E. coli* in the comparison is indicated. Also shown are the data gathered from the Conserved Domains Database (NCBI), indicating potential functions for these genes.

## Figures and Tables

**Table 1:** Strains of genus *Escherichia* used in this study, all with completely sequenced genomes or whole genome shotgun sequences freely available from GenBank. Unless otherwise indicated, they are *Escherichia coli*.

Strain	Type	Sorbose operon	Source/Accession number
CFT073	UPEC (uropathogenic)	+	AE014075
F11	UPEC	+	AAJU00000000
536	UPEC	+	CP000247
UTI89	UPEC	+	CP000243

042	EAEC (enteroaggregative)	−	Sanger Center
B7A	ETEC (enterotoxigenic)	−	AAJT00000000
E24377A	ETEC	−	CP000800

B171	EPEC (enteropathogenic)	−	AAJX00000000
E22	EPEC	−	AAJV00000000
E2348	EPEC	−	Sanger Center
E110019	EPEC	−	AAJW00000000

53638	EIEC (enteroinvasive)	−	AAKB00000000
MG1655	Commensal (Gastrointestinal tract)	−	U00096
HS	Commensal (Gastrointestinal tract)	−	CP000802

SMS-3-5	Environmental	+	CP000970
O157:H7 str. Sakai	EHEC (enterohaemorrhagic)	+	BA000007
O157:H7 EDL933	EHEC	+	AE005174

*Shigella sonnei* 53G	Bacillary Dysentery	+	Sanger Center
*Shigella flexneri* 2a str. 301	Bacillary Dysentery	+	AE005674
*Shigella dysenteriae* Sd197	Bacillary Dysentery	+	CP000034

**Table 2:** Synteny conservation of sets of adjacent colinear genes in 18 sequenced strains of genus *Escherichia*; all these sets of genes are present in *E. coli* CFT073. Those genes which are part of genomic islands, as identified in [18], are marked with an asterisk.

SAC no.	Gene c numbers	No. of genes	Putative function	*E. coli* F11	*E. coli* 536	*E. coli* UTI89	*E. coli* E2348	*E. coli* O42	*E. coli* B171	*E. coli* E24377A	*E. coli* E22	*E. coli* B7A	*E. coli* E110019	*E. coli* 53638	*S. sonnei* 53G	*E. coli* HS	*E. coli* K12 MG1655	*E. coli* O157H7	*E. coli* O157:H7 EDL933	*E. coli* SMS-3-5	*S. flexneri* 2a str. 201	*S. dysenteriae* Sd197
1	c0317 c0323	7	Polysaccharide biosynthesis*	−	−	−	−	−	−	−	−	−	−	−	−	−	−	−	−	−	−	−
2	c0330 c0333	4	Fucose metabolism	+	+	−	+	−	−	−	−	−	−	−	−	−	−	−	−	−	−	−
3	c0409 c0415	7	2,5-diketo-D-gluconic acid metabolism	+	+	+	+	+	−	−	−	−	−	−	−	−	−	−	−	+	−	−
4	c0757 c0765	9	Chorismate biosynthesis	+	+	+	+	−	−	−	−	−	−	−	−	−	−	−	−	−	−	−
5	c1955 c1960	6	PTS system, cellobiose specific	−	−	+	−	−	+	+	+	+	+	+	−	+	−	−	−	+	−	−
6	c3405 c3410	6	PTS system, maltose/glucose specific*	+	+	+	+	−	−	−	−	−	−	−	−	−	−	−	−	+	−	−
7	c3750 c3756	7	5- or 6-carbon sugar metabolism	+	+	+	+	−	−	−	−	−	−	−	−	−	−	−	−	+	−	−
8	c4013 c4018	6	Carbohydrate metabolism	+	+	+	−	−	−	−	−	−	−	−	−	−	−	−	−	−	−	−
9	c4276 c4280	5	PTS system, galactitol specific	−	−	+	−	+	+	−	+	−	−	+	−	−	−	+	+	+	−	−
10	c4481 c4488	8	PTS system, fructose specific	+	+	+	+	−	−	−	−	−	−	−	−	−	−	−	−	−	−	−
11	c4756 c4759	4	PTS system, glucose specific	+	+	+	+	−	−	−	−	−	−	−	−	−	−	−	−	−	−	−
12	c4760 c4780	21	Entner-Doudoroff pathway	+	+	+	−	−	−	−	−	−	−	−	−	−	−	−	−	−	−	−
13	c4828 c4830	3	Shikimate metabolism	+	+	+	+	−	−	−	−	−	+	−	+	+	−	−	−	−	−	−
14	c4924 c4926	3	Citrate metabolism	+	+	+	+	−	−	−	−	−	−	−	−	−	−	−	−	−	−	−
15	c4981 c4987	7	L-sorbose metabolism	+	+	+	+	+	−	−	−	−	−	−	+	−	−	+	+	+	+	−
16	c5020 c5025	6	3-ketoacid metabolism	−	−	+	−	−	−	−	−	−	−	−	−	−	−	−	−	−	−	−
17	c5030 c5041	12	2-oxoglutarate metabolism	+	+	+	+	−	−	−	−	−	−	−	−	−	−	−	−	−	−	−
18	c5298 c5303	6	3-ketoacid metabolism	+	+	+	+	−	+	+	+	+	+	+	−	+	−	−	−	+	−	−
19	c5346 c5351	5	Arginine metabolism	+	+	+	−	−	−	−	−	−	−	−	−	−	−	−	−	−	−	−

**Table 3:** Comparison of SAC No. 15 in CFT073 to the *Klebsiella pneumoniae* L-sorbose degradation operon.

CFT073	CFT073 name	*Klebsiella* name	*Klebsiella*	Protein
gene ID			Locus	Similarity (%)
c4981	Putative oxidoreductase	L-sorbose 1-phosphate reductase	*SorE*	91
c4982	PTS system, mannose-specific	second subunit of EII-Sor	*SorM*	97
	IID component			
c4983	PTS system, mannose-specific	first subunit of EII-Sor	*SorA*	95
	IIC component			

c4984	Putative sorbose PTS component	EIII-B Sor PTS	*SorB*	92
c4985	Putative sorbose PTS component	EIII-F Sor PTS	*SorF*	82
c4986	sorbitol-6-phosphate 2-dehydrogenase	D-glucitol-6-P-Dehydrogenase	*SorD*	92

c4987	Putative transcriptional regulator	Sor regulator	*SorC*	92
	of sorbose uptake and utilization genes			

## Abbreviations

APEC: Avian pathogenic Escherichia coli
EGSS: Escherichia genome sequence set
HGT: Horizontal gene transfer
PMG: Putatively metabolic gene
PTS: Phosphotransferase transport system
UPEC: Uropathogenic Escherichia coli
UTI: Urinary tract infection.
